# Carbon Black-Modified Electrodes Screen-Printed onto Paper Towel, Waxed Paper, and Parafilm M^®^

**DOI:** 10.3390/s17102267

**Published:** 2017-10-03

**Authors:** Stefano Cinti, Vincenzo Mazzaracchio, Ilaria Cacciotti, Danila Moscone, Fabiana Arduini

**Affiliations:** 1 Department of Chemical Science and Technology, University of Rome “Tor Vergata”, Via della Ricerca Scientifica 1, 00133 Rome, Italy; vincenzo.mazzaracchio@uniroma2.it (V.M.); moscone@uniroma2.it (D.M.); 2 Department of Engineering, University of Rome Niccolò Cusano, Via Don Carlo Gnocchi 3, 00166 Rome, Italy; ilaria.cacciotti@unicusano.it (I.C.)

**Keywords:** screen-printed electrodes, carbon black, paper towel, waxed paper, parafilm

## Abstract

Herein, we evaluated the use of paper towel, waxed paper, and Parafilm M^®^ (Heathrow Scientific, Vernon Hills, IL, USA) as alternative substrates for screen-printed sensor manufacturing. Morphological study was performed to evaluate the adhesion of the ink on these uncommon substrates, as well as the morphology of the working electrode. The electrochemical characterization was carried out using ferricyanide/ferrocyanide as redox couple. To enhance the electrochemical properties of the developed sensors, the nanomaterial carbon black was used as nanomodifier. The modification by drop casting of the working electrode surface, using a stable dispersion of carbon black, allows to obtain a sensor with improved electrochemical behavior in terms of peak-to-peak separation, current intensity, and the resistance of charge transfer. The results achieved confirm the possibility of printing the electrode on several cost-effective paper-based materials and the improvement of the electrochemical behavior by using carbon black as sustainable nanomaterial.

## 1. Introduction

By analyzing the major market drivers, restraints, opportunities, and challenges in North America, Europe, and Asia-Pacific areas, the global point-of-care market is expected to reach USD 36.96 Billion by 2021 [1]. In this overall scenario, the electrochemical sensors cover a leading role in this sector as well as in other fields including environmental, food, and security [2,3,4,5,6,7,8,9,10].

For electrochemical device mass-production, printing technology has been widely employed for fabricating customized, miniaturized, and reproducible electrochemical tools at a large scale. Among the different printing techniques, screen-printing is one of the most widely used, because it is a simple and cost-effective technique [11,12,13,14]: it only requires masks, a squeegee, and an oven.

To improve the electroanalytical performance of screen-printed devices, the inks can be modified during or after manufacturing with nanomaterials, such as metal nanoparticles (Au, Pt, Ag, etc.), carbonaceous nanomaterials (graphene, carbon nanotubes, carbon black, etc.), or conductive polymers (polypyrrole, polyaniline polythiophene, etc.). The use of nanomodified inks allows an enhancement of conductivity, defective sites, and high surface-to-volume ratio, boosting the analytical properties of the sensors [15,16,17,18,19]. Among carbonaceous nanomaterials, carbon black (CB) has attracted considerable attention in the scientific community thanks to its outstanding properties in the electrochemical detection of several analytes. Furthermore, it is an inexpensive material that is easily dispersible in inks and solutions to modify the electrodes, and does not require any prior treatment before use [20]. Printed electrodes were modified by drop casting as well as adding CB in the ink, reaching an improvement in terms of reduction of peak-to-peak separation and an increase of peak current intensity using ferro/ferricyanide as electrochemical probe. In addition, the electrochemical study has revealed the best electrochemical behavior using printed electrodes modified by drop casting, probably ascribed to a higher content of CB on the working electrode surface [21]. The printed electrodes modified with CB by drop casting approach have demonstrated their suitability as cost-effective and miniaturized electrochemical sensors for several analytes—including polyphenols, thiols, NADH, and phosphate—to name a few [22,23,24,25].

Although the rush in finding smart nanomaterials for improved analytical methods is still ongoing, the consideration of alternative materials for device manufacturing involves the need of more diverse and uncommon substrates. Paper-based ones have been readily placed on top of the list of this ‘novel’ category of materials, due to their advantages of being of low cost, environmental friendliness, versatility, and ease of application [26,27,28]. Starting with the pioneering works published by research groups headed by Whitesides, Henry, and Crooks [29,30,31,32], paper has displayed its suitability in developing stand-alone tools capable of actively dealing with complex matrices [29,33,34,35]. In 2010, the Whitesides group integrated a chromatographic paper-based device with a commercial electrochemical glucose test strips, further highlighting the use of paper as a novel substrate. By changing the material of the substrate, a cost reduction of $0.5–1.0 per plastic-based strip to $0.014 per paper-based strip was achieved [31]. In addition, we calculated a further 30% savings sensor manufacturing by moving from chromatographic to office paper [11]. These results notably match with the requests made by the WHO, as the cost of $0.5–1.0/strip is impractical for applications in the developing world. As a consequence, novel (and cheaper) solutions need to be developed, going beyond more commonly used substrates such as polyester and alumina.

In recent years, innovations in substrate material and design have been investigated: neoprene wetsuits, mouthguards, cotton fabrics, gloves, stainless-steel pins, etc. represent some of the most diverse (uncommon) substrates used for printed electrode fabrication [36,37,38,39,40,41,42]. Driven by the opportunity in finding materials that are generally utilized in fields that are different from electrochemistry, the list of the uncommon substrates that can find a ‘second life’ in sensor fabrication can be updated.

Herein, we decided to investigate three substrates—namely paper towel, waxed paper, and Parafilm M^®^—that find their application in fields that do not belong to the electrochemistry. To our knowledge, paper towel has not been utilized to develop electrochemical sensors yet. In fact, its high porosity makes this material very effective to adsorb liquids, and this feature can be useful in electroanalysis as well as the exploitation of filter paper (Whatman No. 1). Moreover, paper towel is cheaper than filter paper (<90%) and it would be consistent with the lowering of mass-scale production of sensors. Similar to the previous uncommon material, Parafilm^®^ and waxed paper are very well-known materials for packaging, that can be employed for developing sensing platforms as well. In fact, one can image printing a sensor directly onto a packed product for evaluating the quality of the product itself. Based on these motivations, carbon electrodes have been screen-printed onto paper towel, Parafilm^®^, and waxed paper, and they have been morphologically and electrochemically examined. In addition, the modification with cost-effective CB by drop casting onto the working electrode area has been evaluated, highlighting a clear improvement of the electrochemical properties of the nanomodified printed-strips.

## 2. Experimental Section

### 2.1. Chemicals and Equipments

Potassium ferricyanide (K_3_Fe(CN)_6_), potassium ferrocyanide (K_4_Fe(CN)_6_), hydroquinone, hexaammineruthenium trichloride Ru(NH_3_)_6_Cl_3_, potassium chloride (KCl), and *N*,*N*-Dimethylformamide (DMF) were purchased from Sigma Aldrich (St. Louis, MO, USA). Carbon black CB-N220 powder was kindly gifted by Cabot Corporation (Ravenna, Italy). All solutions were prepared using distilled water. Experiments of cyclic voltammetry were performed by using a portable potentiostat EmStat^3^ (Palmsens, The Netherlands). Electrochemical Impedance Spectroscopy measurements were performed by using a PalmSens 3 potentiostat (Palmsens, The Netherlands), and the fitting of the data was obtained by using Z-view software (Scribner Associates, Inc., Southern Pines, NC, USA; www.scribner.com). All the EIS measurements were carried out with an AC voltage of 0.05 V in a frequency range comprised between 10,000 and 0.1 Hz. The morphology was investigated by observation with field emission scanning electron microscopy (FEG-SEM, Leo Supra 35, Cambridge, UK).

### 2.2. Screen-Printed Electrodes

**Figure 1:**
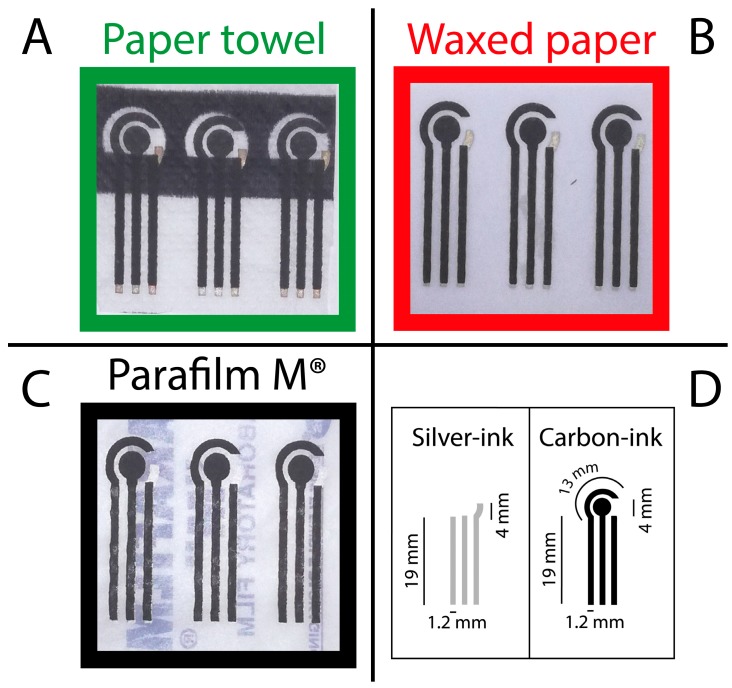
Photographs of electrodes screen-printed on (**A**) paper towel; (**B**) waxed paper; and (**C**) Parafilm^®^; (**D**) Dimensions of the conductive tracks that have been screen-printed.

The three-electrode system was manually screen-printed using a squeegee and two masks (mask 1 and mask 2), Figure 1. Firstly, mask 1 was used to print the connections and the pseudo-reference electrode by using an Ag/AgCl ink (Electrodag 477 SS) and, successively, mask 2 was used to screen-print carbon ink (Electrodag 421) that served as the working and counter electrodes. After each printing step, the SPEs were cured at 60 °C for 30 min, in order to prevent the damage of the substrates, namely paper towel, waxed paper, and Parafilm M^®^. All the conductive inks were purchased from Acheson (Italy). In a single sheet of the chosen substrate, eight electrodes were screen-printed. The diameter of the working electrode was 4 mm (Figure 1D). The resistance of the three substrates was measured with a multimeter tester (ISO-TECH, IDM 62T) resulting equal to 0.25 ± 0.04 kΩ, 2.70 ± 0.05 kΩ, and 4.25 ± 0.40 kΩ, respectively for paper towel, waxed paper, and Parafilm M^®^.

### 2.3. Preparation of CB Dispersion

CB powder was used to produce dispersion at a concentration of 1 mg/mL in Dimethylformamide (DMF): water (1:1 v/v) mixture, and then sonicated for 60 min at 59 kHz. The dispersion was stable for more than one month if stored in dark condition and at room temperature.

### 2.4. Modification of the Screen-Printed Electrodes

The dispersion was used to modify screen-printed electrodes (SPEs) via drop casting. Briefly, a tiny volume (2 μL) of the CB dispersion was drop cast onto the working electrode surface in a single step.

### 2.5. Electrochemical Measurements

The electrochemical cell for the SPEs onto waxed paper and Parafilm M^®^ was a 100 μL-drop, while the measurements for the paper towel-based SPE were performed exploiting the paper porosity, which was impregnated with 10 μL of solution.

## 3. Results and Discussion

### 3.1. SEM Characterization of the Substrates

**Figure 2:**
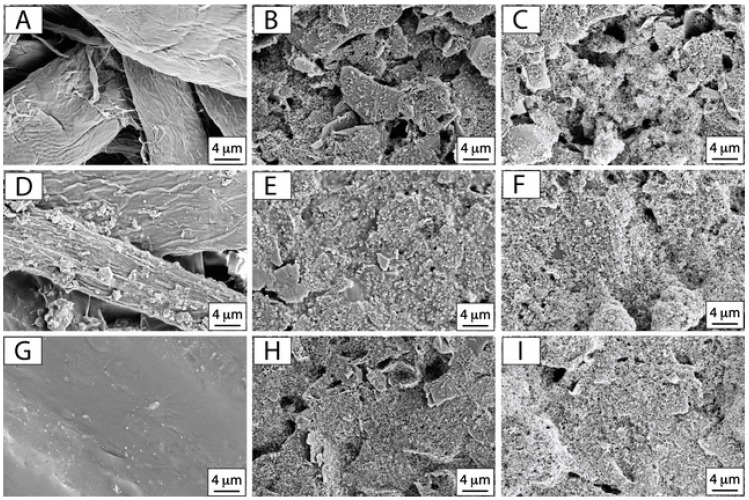
SEM micrographs of: pristine (**A**) paper towel, (**D**) waxed paper, (**G**) Parafilm M^®^; SPEs onto (**B**) paper towel, (**E**) waxed paper, (**H**) Parafilm M^®^; CB modified SPEs onto (**C**) paper towel, (**F**) waxed paper, (**I**) Parafilm M^®^. Acceleration voltage: 5 keV, magnification 10 kx.

Prior to investigate the electrochemical properties of the chosen platforms, scanning electron microscopy (SEM) observation was carried out. Paper towel, waxed paper, and Parafilm M^®^ were firstly examined before and after the screen printing process and the SPEs modification with CB dispersion, as reported in Figure 2.

As expected, SEM micrographs reported in Figure 2A,D,G highlight the morphological diversity of the substrates that have been investigated in this study. Paper towel is a very adsorbent tissue and its cellulosic structure appears extremely wrinkled and the fibers seem widely distributed (Figure 2A). By observing the waxed paper, the roughness is still relevant but lower if compared to the paper towel (Figure 2D). In fact, waxed paper was obtained by utilizing office paper that was impregnated with melted paraffin, making it more hydrophobic. The presence of the cellulosic fibers due to the office paper is evident, but their density appears higher than the paper towel (Figure 2D) due to the paraffin impregnation and coverage. The last substrate that was taken into account is Parafilm M^®^ that consists in a plastic paraffin film. Its morphology appears totally different with respect to the previous substrates, being compact and characterized by relevant smoothness (Figure 2G). These differences might lead to different adhesion of the conductive-ink during the screen printing process. In order to evaluate the influence of the substrate microstructure on the ink deposition, electrodes screen-printed on the investigated supports were also observed at SEM. From the SEM micrograph of the SPE on paper towel (Figure 2B), it is evident that the graphite sheets of the carbon-based ink are not evenly distributed on the surface, reflecting the roughness and inhomogeneity of the underlying substrate composed of randomly oriented cellulosic fibers. By observing the other substrates in Figure 2E,H, respectively for waxed paper and Parafilm M^®^, graphite sheets seem to be homogeneously distributed on the surface of the substrates (voids are limited), leading to a continuous and compact ink layer. These features can be ascribed to the different nature of the used substrates. The waxed paper and Parafilm M^®^ hydrophobic nature could allow the ink, that is mainly composed by hydrophobic components (polymers, solvents), to strongly adhere onto their surfaces and to a better impregnation, whereas the hydrophilic and wrinkled microstructure of paper towel could lead to a more inhomogeneous distribution of the ink on its surface and to the presence of numerous voids between the more evident and emerged graphite sheets.

In the case of CB modified SPEs (Figure 2C,F,I), the drop casting of CB nanoparticles allowed a complete and uniform coverage of the below screen printed electrode, following the morphology and microstructure of the underlying working electrode. It is also possible to observe the presence of numerous cauliflower-like agglomerates, as expected on the basis of previous findings, leading to a higher surface roughness [24].

### 3.2. Cyclic Voltammetry Experiments of the SPEs

**Figure 3:**
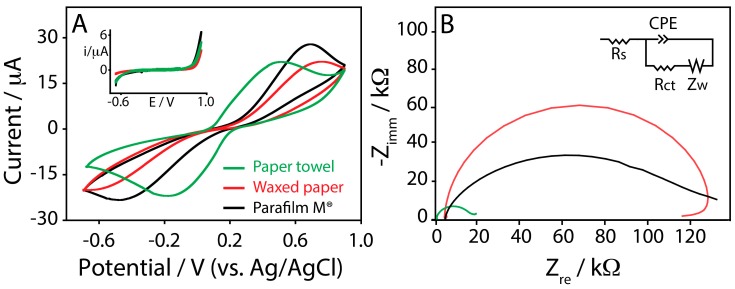
(**A**) Cyclic voltammograms performed in 2 mM ferricyanide/ferrocyanide (Fe(CN)_6_^3−^/Fe(CN)_6_^4−^), prepared in 100 mM potassium chloride, at a scan rate of 0.05 V/s, for (green line) paper towel, (red line) waxed paper, and (black line) Parafilm M^®^. Inset: cyclic voltammograms performed in 100 mM potassium chloride, at a scan rate of 0.05 V/s, for (green line) paper towel, (red line) waxed paper, and (black line) Parafilm M^®^; (**B**) Complex plane impedance plots at open circuit potential, using a 2 mM ferricyanide/ferrocyanide in 0.1 M KCl for (green line) paper towel, (red line) waxed paper, and (black line) Parafilm M^®^. Inset: Randles circuit.

To evaluate the suitability of these novel platforms to be utilized as electrochemical sensors, cyclic voltammetry experiments were performed. To do this, the well-known redox couple based on ferricyanide/ferrocyanide (Fe(CN)_6_^3−^/Fe(CN)_6_^4−^) was adopted in order to obtain information about the electron transfer process at the SPE. All the typologies of the SPEs were interrogated in a solution containing 2 mM of the redox couple by varying the scan rate from 0.05 to 1 V/s. Figure 3A displays the cyclic voltammograms obtained, taking into account a scan rate of 0.05 V/s, in order to provide a quick comparison among the investigated platforms.

The peak-to-peak separation (∆E) was calculated using 0.05 V/s as the scan rate, and, as it can be noted, the resulting peak separation was different among the investigated substrates. It was 0.66 ± 0.07, 1.38 ± 0.13, and 1.33 ± 0.15 V (Table 1), respectively to paper towel, waxed paper, and Parafilm M^®^ SPEs. ∆E provides a qualitative estimation of the electron transfer rate due to the redox process at the electrode surface, and it appears evident how the different substrates are able to influence the electrode reaction kinetics. The reason could be due to the different ink/substrate interfaces used during the SPEs production and to different obtained ink adhesion. Even if the hydrophobic nature of waxed paper and Parafilm M^®^ should assure a more favorable adhesion of the ink with respect to the paper towel, the wrinkled structure of the paper towel would be responsible for a more effective ink deposition within the cellulose network, changing the working electrode surface behavior. The response of bare SPEs and the CB-modified ones was evaluated in presence of the redox couple ferro/ferricyanide using CV over a scan range comprised between 0.05 and 1 V/s. The current peaks increased linearly in both cases with the square root of the scan rate, indicating a semi-infinite linear diffusion-controlled current (Figure S1, Supporting Information). To better understand this result, SPEs were tested using electrochemical impedance spectroscopy, because this technique can provide useful information on the impedance changes of the electrode surface by measuring the value of electron transfer resistance (Rct) [43]. The Rct is estimated by measuring the diameter of the semicircle present at the high frequency region, giving information about the difficulty of electron transfer of ferro/ferricyanide redox probe between the solution and the electrode. Electrochemical impedance spectroscopy was performed with paper towel, waxed paper, and Parafilm M^®^ SPEs at open circuit potential (OPC). The fitting of spectra was carried out using the equivalent electrical circuit (Randles circuit) showed in Figure 3B (inset), comprising the electrolyte resistance, Rs, in series with a parallel combination of Rct (interfacial charge transfer resistance), Zw (diffusion of the analytes in solution and corresponding to Warburg impedance straight line of the curves), and CPE (Constant Phase Element). In Table 2 the values of Rs, Rct, and CPE for each type of the sensor are reported. In the case of paper towel SPE, a lower Rct was observed with respect to waxed paper and Parafilm M^®^ SPEs, confirming the data obtained using cyclic voltammetry technique.

**Figure 4:**
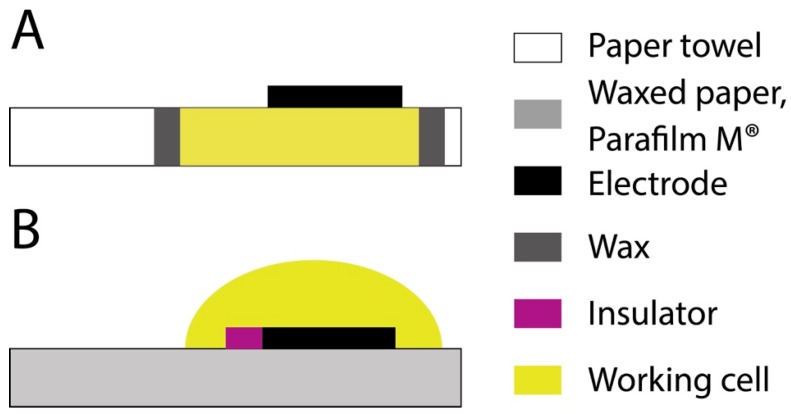
Experimental configurations of electrodes that have been screen-printed onto (**A**) paper towel and (**B**) waxed paper and Parafilm M^®^.

In addition, in the case of paper-towel a lower value of Rs was also observed, probably due to the different diffusion layers in the case of paper towel SPE when compared with waxed paper and Parafilm M^®^ SPEs. Indeed, in the last cases, the solution was confined in a drop onto the working electrode surface due to the hydrophobic character of the whole electrochemical cell, while in the case of paper-towel SPE, the solution went through the cellulosic network, as schematized in Figure 4.

The constant phase element (CPE) determination was necessary due to the non-homogeneous surface of the working electrode and it is modeled as a non-ideal capacitor of capacitance C and roughness/non-uniformity factor α. The lower value of α was observed in the case of Parafilm M^®^ SPEs, suggesting a rougher electrode surface with respect to waxed paper and paper towel SPEs. Also, the CPE value confirms how the substrate can influence the electrochemical behavior of the working electrode printed on different materials.

### 3.3. Improving the Electrochemical Performances of the SPEs with CB

**Figure 5:**
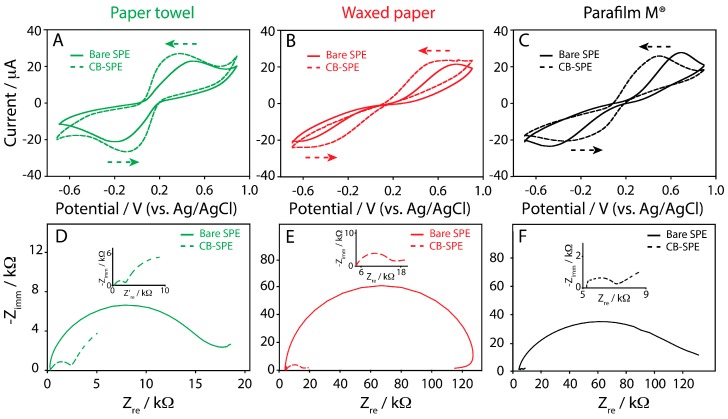
Cyclic voltammograms performed in 2 mM ferricyanide/ferrocyanide (Fe(CN)_6_^3−^/Fe(CN)_6_^4−^), prepared in 100 mM potassium chloride, at a scan rate of 0.05 V/s, using: (**A**) bare (green solid line) and CB-modified (green dashed line) SPE onto paper towel; (**B**) bare (red solid line) and CB-modified (red dashed line) SPE onto waxed paper; (**C**) bare (black solid line) and CB-modified (black dashed line) SPE onto Parafilm M^®^. Complex plane impedance plots at open circuit potential using a 2 mM ferricyanide/ferrocyanide in 100 mM potassium chloride for: (**D**) bare (green solid line) and CB-modified (green dashed line) SPE onto paper towel; (**E**) bare (red solid line) and CB-modified (red dashed line) SPE onto waxed paper; (**F**) bare (black solid line) and CB-modified (black dashed line) SPE onto Parafilm M^®^. Insets of Figure 5D–F highlight the complex plane impedance plot for CB-SPE.

As widely reported in our recent works [20,21,22,23,24,25], and by other influent research groups, as well as Pumera’s group [44] and Compton’s group [45,46], CB is capable to strongly influence the performance of electrochemical platforms. In this work, all the SPEs were modified with a 1:1 DMF/water dispersion of CB. The platforms were drop cast with a 2-μL volume of the CB dispersion and, after the solvent evaporation (2 min at 37 °C), cyclic voltammetry experiments were carried out. The redox couple Fe(CN)_6_^3−^/Fe(CN)_6_^4−^ was used as the working solution. As reported in Figure 5, a net improvement of the electrochemical response was observed.

**Figure 6:**
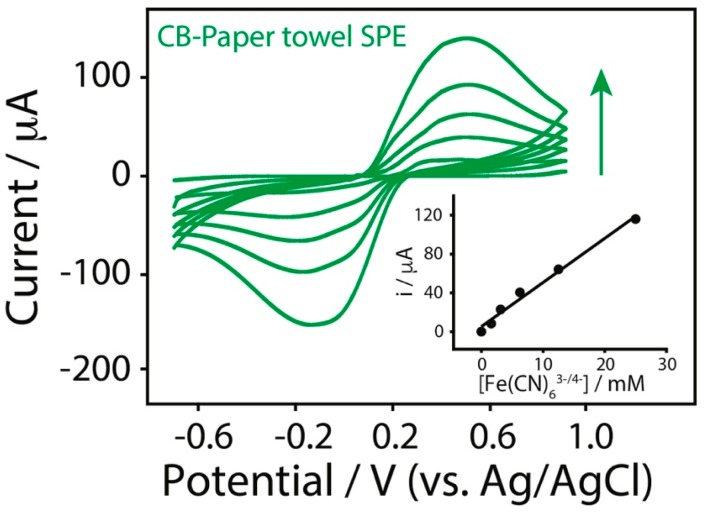
Cyclic voltammograms of CB-paper towel SPE in presence of ferri/ferrocyanide concentrations comprised between 1.5 and 25 mM in 100 mM potassium chloride (scan rate of 0.05 V/s). All the experiments were performed by adding 10 μL of the solution to analyze on the back of the testing area. Inset: calibration plot of the tested ferri/ferrocyanide concentrations.

The modification of the SPEs with CB dispersion led to an important improvement in terms of peak-to-peak separation, which is consistent with a better electron transfer, if compared to the bare SPEs. In Table 1 the anodic currents and peak-to-peak separations, obtained at a scan rate of 0.05 V/s for different substrates, are reported. Table 1 also contains the equation of the curves related to the CB-modified SPEs used for the detection of increasing concentration of ferri/ferrocyanide (1.5–25 mM). In addition, an example of well-defined voltammetric response to different ferri/ferrocyanide concentrations up to 25 mM is shown in Figure 6, and all the curves have been obtained with one-shot CB-modified paper towel SPE.

These data suggest that, while peak currents were not strongly affected in intensities, both comparing the different substrates and the bare SPEs with the CB-modified SPEs (a slight increase of the current was observed), the presence of the nanomodifier led to relevant decrements in the ∆E values calculated at 0.05 V/s. Indeed, ∆E decreased from 0.66 ± 0.07 to 0.45 ± 0.05 V, from 1.38 ± 0.13 to 0.84 ± 0.07, and from 1.33 ± 0.15 to 0.76 ± 0.08 V, respectively for paper towel, waxed paper, and Parafilm M^®^ CB-SPEs.

The data obtained by using our sensors and electrochemical impedance spectroscopy as technique were reported in Figure 5D–F. The presence of CB as a nanomodifier strongly decreases the Rct values, as reported also in Table 2, confirming the better electron transfer with respect to the bare electrode, as well as the data resulted from cyclic voltammetry experiments.

Particularly, the lowest Rct values were observed in the case of paper towel and Parafilm M^®^ SPEs. Evaluating CPE values, the presence of nanomodifiers allows the decrease of α, confirming the increase of the roughness, as expected and supported by SEM observation (Figure 2C,F,I).

Even if not displaying outstanding features, the use of CB as a working electrode modifier is able to make the same platforms more suitable to be used in the development of electroanalytical devices.

In addition to the negatively charged ferri/ferrocyanide redox couple, the three investigated electrochemical platforms, successively enhanced with CB, were interrogated in the presence of two other redox probes—the neutral hydroquinone (HQ) and the positively charged hexaammineruthenium (Ru(NH_3_)_6_^3+^)—where it was observed that the charge of the redox probe affects the electrochemical response of the printed sensors (Figures S2–S4, Supporting Information).

## 4. Conclusions

The main goal of this work was to investigate novel substrates that could serve in electrochemical sensors manufacturing. Three uncommon substrates—namely paper towel, waxed paper, and Parafilm M^®^—were investigated by means of morphological and electrochemical approaches. From the comparison of the different substrates, the best electrochemical response was registered in the case of electrodes screen printed on paper towel, probably due to its higher porosity, as well as different diffusion of solution; however, a further deeper investigation is required. Indeed, a lower ∆E value (calculated using 0.05 V/s scan rate) was measured: 0.66 ± 0.07 V for SPEs on paper towel vs 1.38 ± 0.13 V and 1.33 ± 0.15 V for waxed paper and Parafilm M^®^ SPEs, respectively. Moreover, since the fabricated SPEs were not so efficient in terms of electrochemical performances, SPEs were modified with the cost-effective nanomaterial, carbon black, by drop casting. In the case of cyclic voltammetry experiments, using ferricyanide/ferrocyanide as redox couple, a net decrease of the peak-to-peak separation compared to the bare SPEs was observed, that is consistent with the improvement of the platforms.

Thus, in conclusion, it has been demonstrated that uncommon substrates—namely paper towel, waxed paper, and Parafilm M^®^—can be used as supports to print the electrodes when modified with carbon black. In addition, our results also corroborate the importance of the substrate intrinsic characteristics in the printed electrochemical sensor development: porosity, roughness, and composition of the substrate can lead to completely different results in terms of ink adhesion and the related electrochemical behavior.

## Figures and Tables

**Table 1:** Summary of electrochemical parameters for SPEs fabricated onto paper towel, waxed paper, and Parafilm M^®^, before (SPE) and after (CB-SPE) the modification with 2 μL of a CB dispersion.

Substrate for SPE Fabrication	SPE (i/μA) *	SPE (∆E/V) **	CB-SPE (i/μA) *	CB-SPE (∆E/V) **	Equation Using CB-SPE
Paper towel	16.7 ± 2.0	0.66 ± 0.07	18.0 ± 2.7	0.45 ± 0.05	y = 5.2 + 4.5 x
					*R*^2^ = 0.9877
Waxed paper	11.2 ± 1.8	1.38 ± 0.13	13.3 ± 2.0	0.84 ± 0.07	y = 2.9 + 2.3 x
					*R*^2^ = 0.9881
Parafilm M^®^	17.1 ± 2.4	1.33 ± 0.15	17.4 ± 1.9	0.76 ± 0.08	y = 1.5 + 3.2 x
					*R*^2^ = 0.9781

* Anodic current; ** ∆E at 0.05 V/s.

**Table 2:** Summary of the EIS parameters for SPEs fabricated onto paper towel, waxed paper, and Parafilm M^®^, before (SPE) and after (CB-SPE) the modification with 2 μL of a CB dispersion.

Substrate	Rs (Ω)	Rct (Ω)	α (CPE)
Paper towel SPE	284 ± 9	15,670 ± 300	0.94
Paper towel CB-SPE	294 ± 7	2583 ± 61	0.73
Waxed paper SPE	6513 ± 36	118,700 ± 500	0.97
Waxed paper CB-SPE	4542 ± 15	11562 ± 93	0.75
Parafilm M^®^ SPE	3947 ± 57	110,700 ± 1700	0.76
Parafilm M^®^ CB-SPE	4988 ± 20	2283 ± 34	0.64

## Supplementary Materials

The following are available online at http://www.mdpi.com/1424-8220/17/10/2267/s1, Figure S1: Cyclic voltammetric study in presence of ferri/ferrocyanide varying the scan rate, Figure S2: Cyclic Voltammetry study in presence of hydroquinone and hexaammineruthenium, Figure S3: Cyclic voltammetry study in presence of hydroquinone varying the scan rate, Figure S4: Cyclic voltammetry study in presence of hexaammineruthenium varying the scan rates.
