# Supplementary material for: Carbon Black-Modified Electrodes Screen-Printed onto Paper Towel, Waxed Paper, and Parafilm M®
Source: Sensors (Basel). 2017 Oct 3;17(10):2267. doi: 10.3390/s17102267 (PMC5676850; doi:10.3390/s17102267)
Supplement: Supplementary file 1 [file sensors-17-02267-s001.pdf]

Supplementary Materials: Carbon Black-Modified Electrodes Screen-Printed onto Paper Towel, Waxed Paper, and Parafilm M®

Stefano Cinti, Vincenzo Mazzaracchio, Ilaria Cacciotti, Danila Moscone, and Fabiana Arduini

1. Cyclic Voltammetric Study in Presence of Ferri/Ferrocyanide Varying the Scan Rate

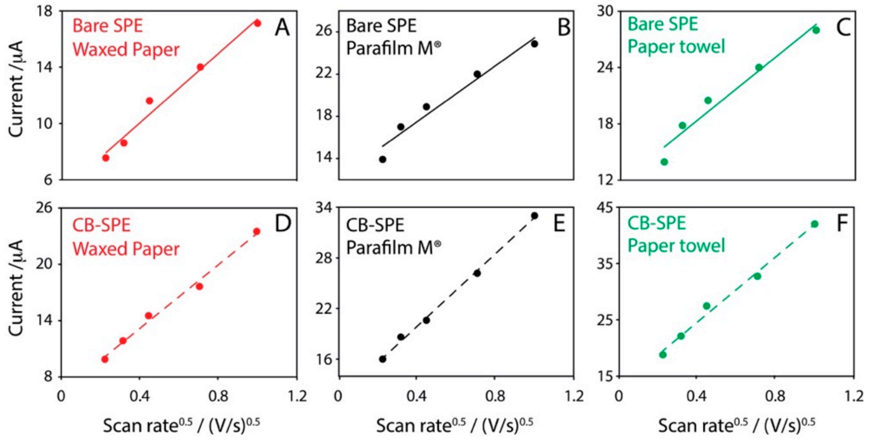

Figure S1. Current vs. square root of scan rate for bare SPEs onto (**A**) waxed paper, (**B**) Parafilm M ®, and (**C**) paper towel, and CB-modified SPEs onto (**D**) waxed paper, (**E**) Parafilm M ®, and (**F**) paper towel. CV measurements in presence of 2 mM ferro/ferricyanide in the scan rate range comprised between 0.05 and 1 V/s.

2. Cyclic Voltammetry Study in Presence of Hydroquinone and Hexaammineruthenium

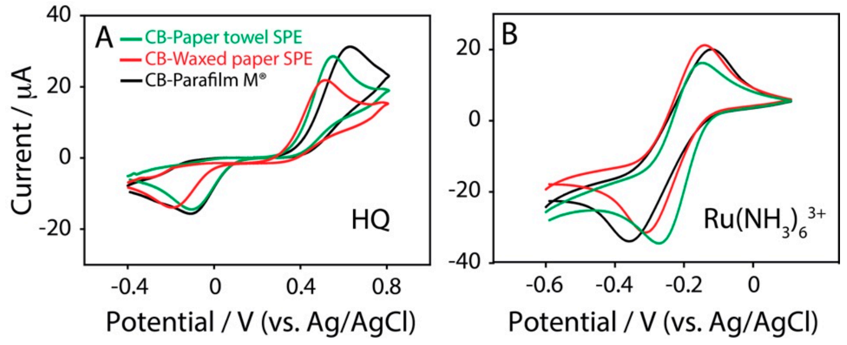

Figure S2. Cyclic voltammograms performed in (**A**) 2 mM hydroquinone and (**B**) 2 mM hexaammineruthenium, prepared in 100 mM potassium chloride, at a scan rate of 0.05 V/s, for CB-modified SPEs onto (green line) paper towel, (red line) waxed paper, and (black line) Parafilm M®.

The different supports have been tested in presence of the two redox species, by varying the scan rate in a range comprised between 0.05 and 1 V/s. Figure S3 and S4 display the electrochemically characterization in presence of 2 mM hydroquinone and 2 mM hexaammineruthenium, respectively.

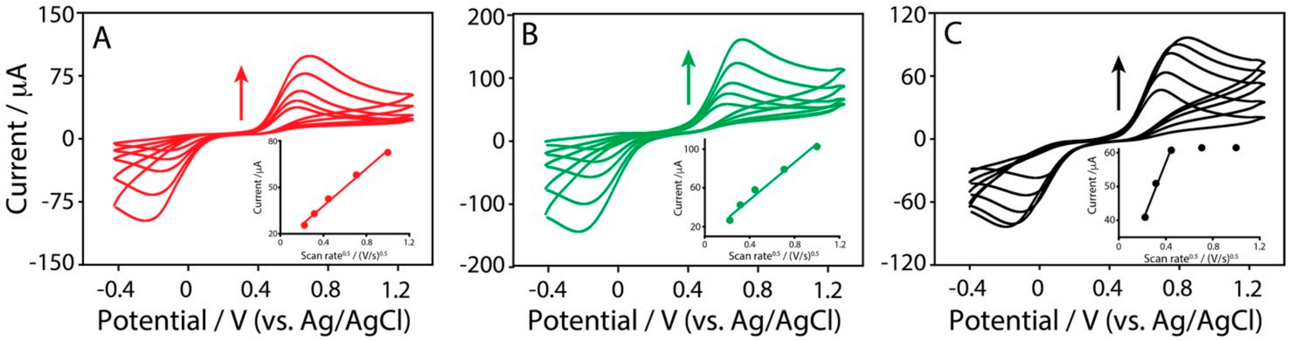

Figure S3. Cyclic voltammograms performed in 2 mM hydroquinone in 100 mM potassium chloride, by varying the scan rate from 0.05 to 1 V/s, using CB-modified SPEs onto **(A)** waxed paper, (**B**) paper towel, and (**C**) Parafilm M®. Insets: (current) vs (scan rate)$^{0.5}$.

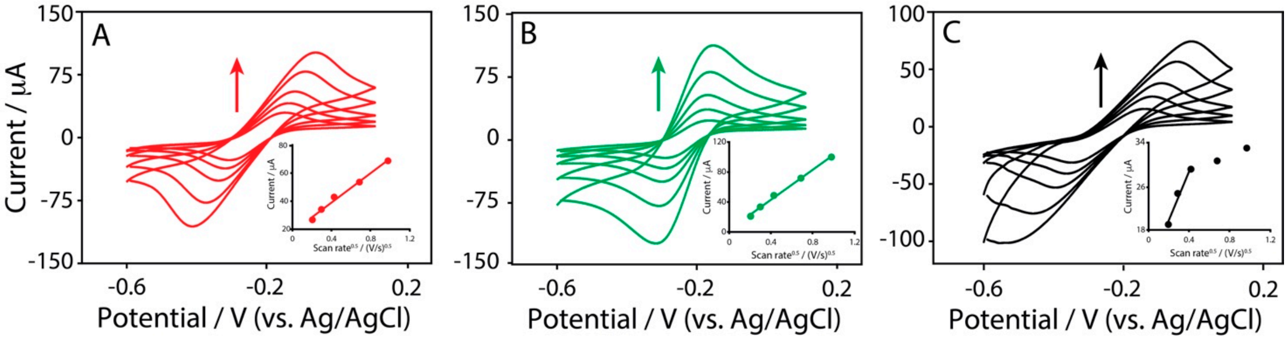

Figure S4. Cyclic voltammograms performed in 2 mM hexaammineruthenium in 100 mM potassium chloride, by varying the scan rate from 0.05 to 1 V/s, using CB-modified SPEs onto (**A**) waxed paper, (**B**) paper towel, and (**C**) Parafilm M®. Insets: (current) vs (scan rate)$^{0.5}$.

For each of the SPEs tested, anodic currents have been linearly correlated with the square root of the scan rate utilized during the single experiment. As displayed in the insets of Figures S3 and S4, the mass transfer is diffusion controlled, in agreement with the Randles-Sevcik equation. While for low scan rates all the SPEs behaved similarly in terms of current vs (scan rate)$^{0.5}$- linearity, when high scan rates are interrogated (>0.5 V/s) the electrodes printed on Parafilm M® showed loss of peaks symmetry and also a loss of the diffusion controlled mechanism.
